# Policy Series: Caregiving in the US 2025: Emerging Themes and Actionable Policy and Practice Solutions

**DOI:** 10.1093/geroni/igaf122.1186

**Published:** 2025-12-31

**Authors:** Selena Caldera, Rita Choula

## Abstract

Every five years, the National Alliance for Caregiving and AARP’s Caregiving in the US (CGUS) survey provides a snapshot of family caregivers, their care recipients, care tasks, help with caregiving, supports received in health systems, and the work and financial impacts of caregiving. This symposium presents emerging themes from the 2025 CGUS survey and highlights actionable policy and practice solutions in each area. Family caregivers are now providing help with more activities of daily living and instrumental activities of daily living than in the past, yet few receive training on how to perform these tasks. Presenters 1 and 2 discuss these findings and the Caregiver Training Services (CTS) Codes introduced for Medicare providers in 2024. They share qualitative work examining provider experiences supporting caregivers in health systems and offer a practical guide for using the new CTS codes. Caregivers report greater availability of supportive workplace policies but continue to struggle balancing work and care responsibilities. Presenter 3 overviews these new findings and caregivers’ ongoing struggle to balance work and care responsibilities despite increased support at work. While many caregivers experience financial pressures due to caregiving, certain groups disproportionately face these challenges. Presenter 4 summarizes the financial impact of caregiving, focusing on younger caregivers and other groups most affected by financial impacts. Presenters 3 and 4 highlight progress in introducing policies to support working caregivers and innovative new policy solutions to support caregivers financially. Presenter 5 shares the newest innovation in CGUS: state-level data on caregivers and their experiences.

